# Cutting Edge Endogenous Promoting and Exogenous Driven Strategies for Bone Regeneration

**DOI:** 10.3390/ijms22147724

**Published:** 2021-07-20

**Authors:** Iratxe Macías, Natividad Alcorta-Sevillano, Arantza Infante, Clara I. Rodríguez

**Affiliations:** 1 Stem Cells and Cell Therapy Laboratory, BioCruces Bizkaia Health Research Institute, Cruces University Hospital, Plaza de Cruces S/N, 48903 Barakaldo, Spain; IRATXE.MACIASGARCIA@osakidetza.eus (I.M.); NATIVIDAD.ALCORTASEVILLANO@osakidetza.eus (N.A.-S.); 2 University of Basque Country UPV/EHU, 48940 Leioa, Spain

**Keywords:** MSCs, bone regeneration, tissue engineering, scaffold, composite, hydrogel, cell therapy

## Abstract

Bone damage leading to bone loss can arise from a wide range of causes, including those intrinsic to individuals such as infections or diseases with metabolic (diabetes), genetic (osteogenesis imperfecta), and/or age-related (osteoporosis) etiology, or extrinsic ones coming from external insults such as trauma or surgery. Although bone tissue has an intrinsic capacity of self-repair, large bone defects often require anabolic treatments targeting bone formation process and/or bone grafts, aiming to restore bone loss. The current bone surrogates used for clinical purposes are autologous, allogeneic, or xenogeneic bone grafts, which although effective imply a number of limitations: the need to remove bone from another location in the case of autologous transplants and the possibility of an immune rejection when using allogeneic or xenogeneic grafts. To overcome these limitations, cutting edge therapies for skeletal regeneration of bone defects are currently under extensive research with promising results; such as those boosting endogenous bone regeneration, by the stimulation of host cells, or the ones driven exogenously with scaffolds, biomolecules, and mesenchymal stem cells as key players of bone healing process.

## 1. Introduction

Bone, a dynamic natural composite, is constantly remodeled by fine-tuned bone formation and bone resorption processes, carried out by osteoblasts and osteoclasts, respectively, throughout an individual’s lifespan [1]. Bone tissue usually presents self-repairing ability after an injury, regaining the damaged part its original structure and mechanical strength. In fact, bone fracture healing relying on mesenchymal stem cells (MSCs) derived osteoblasts performance, can occur through two different mechanisms: intramembranous (involved in the formation of flat bones such as skull bones and clavicles) and endochondral (in long bones such as femur and tibia) bone formation. While the intramembranous ossification directly forms the bone from MSCs that are differentiated into osteoblasts, for endochondral bone formation, there are two key players required; the presence of cartilage, and the vascularization process [2,3]. Indeed, angiogenesis (the formation of new blood vessels from pre-existing ones) is a key component in bone repair, since blood vessels bring oxygen and nutrients to the regenerating tissue [4]. Moreover, blood vessels supply inflammatory cells, cartilage, and bone precursor cells to reach the injury site, along with the ions necessary for mineralization in a later phase [5]. 

However, bone loss (such as osteoporosis), bone defects of a critical size (defined as those that will not heal spontaneously within a patient’s lifetime [6,7]), lack of vascularization, infections and tumors remain key challenges for successful bone healing [8] and require clinical intervention. In fact, osteoporosis, a highly prevalent bone disease associated to aging and characterized by bone fragility, represents a considerable socio-economic problem whose incidence is irremediably increasing as a consequence of aging of the population. In 2010 there were estimated to be 158 million individuals at high fracture risk worldwide, and demographic shifts mean that this figure is likely to double by 2040 [9]. Current clinical approaches to treat bone defects mainly contemplate natural bone grafts, which although effective present several serious limitations [10,11]. Therefore, alternatives focused on developing synthetic bone tissue surrogates, with scaffolds as central players, are being explored in order to circumvent these disadvantages [12]. An ideal scaffold with bone regeneration purposes should mimic the extracellular matrix (ECM) of natural bone tissue, providing the cells an adequate substrate for adhesion, proliferation, migration, and differentiation [13]. This ECM-cell interaction (including osteoblasts, endothelial cells (EC) and immune cells) will direct the cells fate and control bone repair and regeneration [14]. Taking the ECM interactions into account, the scaffold must fulfill a series of requirements to ensure a proper bone regeneration: first, the scaffold must induce the recruitment and osteogenic differentiation of MSCs in order to form bone (osteoinductivity), and it has to be capable of supporting bone formation (osteoconductivity). Second, the optimal scaffold should ensure the development of vascular networks to warrant a positive suitable microenvironment for tissue engineering [15]. Osteointegration is finally needed, in which the stable anchorage of the scaffold is achieved by direct bone-to-implant contact [16]. 

Until today, numerous strategies have been developed with the purpose of improving bone tissue regeneration. The current review will summarize recent approaches addressing this aim, either by promoting the mobilization and differentiation of endogenous bone progenitor cells or by treating bone defects with the exogenous addition of different agents (scaffolds, biomolecules, MSCs).

## 2. Strategies Promoting Bone Healing through an Endogenous Response

Bone, a heterogeneous composite material, involves living cells embedded in a mineralized ECM consisting of inorganic and organic phases in addition to water [17]. While the inorganic phase is composed of a combination of calcium and phosphorus salts, (predominantly in the form of hydroxyapatite (HA; Ca_10_(PO_4_)_6_(OH)_2_), the organic fraction comprises mainly collagen type I, and other non-collagenous proteins. The amount, proper arrangement, and characteristics of each of these components (quantity and quality) define the properties of bone. However, the relative amount and characteristics of each of these phases present in a given bone varies with age [18], location (bone tissue composition varies across anatomic sites in the proximal femur and the iliac crest), gender [19], and health status [20]. One of the main challenges of bone tissue engineering is to develop scaffolds using materials that emulate the properties of the native bone, composed of unidirectionally aligned collagen fibrils, and densely mineralized with HA crystals. 

### 2.1. Additive-Free Scaffolds: Calcium Phosphate-Based Scaffolds

Osteoblasts begin the mineralization process with the secretion of vesicles filled with amorphous calcium phosphate (ACP), a calcium phosphate (CaP) precipitate of variable composition that acts as a precursor of mineralized bone matrix. ACP granules are deposited into the collagen fibrils, which subsequently, at a pH above 9, are transformed into HA crystals, resulting in the matured, mineralized collagen matrix [21]. However, between 7 and 9 pH range, ACP is transformed into octacalcium phosphate (OCP) phase that, in turn, spontaneously converts to stable HA. Depending on the chemical conditions of the environment (pH and ion concentrations) other CaP phases can be found such as dicalcium phosphate dihydrate (brushite) or tricalcium phosphate (TCP) phases. Therefore, the use of CaP-based scaffolds with different formulations (HA, α- and β-TCPs, OCP, ACP, biphasic CaPs or a mixture of HA and β-TCP at varying ratios) have been considered an ideal artificial bone substitute. Their success relies on their biocompatibility, bioactivity, osteoinductivity and osteoconductivity abilities [22,23]. The mechanism behind the osteoinductive capacity of CaP-based composites has been addressed by a proteomic analysis, which revealed the implication of plasma cell glycoprotein 1 (PC-1), encoded by the ectonucleotide pyrophosphatase/phosphodiesterase 1 gene (*ENPP1*), which regulates the mineralization process by hydrolyzing adenosine triphosphate into adenosine monophosphate and pyrophosphate (PPi) [24]. In fact, only the cells in direct contact with CaP ceramics showed an increase in the expression of *ENPP1* and PC-1 synthesis when compared to non-osteoinductive ceramics, together with other osteogenic markers (bone morphogenetic protein 2 (BMP-2) and Osteopontin), but without affecting the expression of alkaline phosphatase (ALP) [25]. Extracellular PPi levels are key in regulating the mineralization process; thus, PPi is hydrolyzed by ALP to yield inorganic phosphate, a precursor of bone mineral, but excess PPi inhibits bone mineralization and soft tissue calcification by binding to nascent HA crystals, preventing them from continuing to grow. The increased production of PPi by PC-1 in cells cultured in CaP-based scaffolds negatively regulates tissue mineralization, which draws attention to the modulation of *ENPP1* expression as a regulatory response to CaP-induced human MSCs (hMSCs) differentiation to restrict further mineralization [24]. Moreover, the fact that *EPNN1*/PC-1 over-expression occurs only in cells with direct contact with the ceramic, suggests that a chemically-driven process was occurring at the surface involving the exchange of calcium and phosphate ions between the medium and the material. Thus, in this type of intrinsic osteoinduction, which is also known as material induced heterotropic ossification, calcium and phosphate ions precipitate at the surface of the scaffold, forming an apatite layer generating a local depletion of these ions that triggers cellular differentiation into osteogenic lineage [26]. 

Several studies have underlined the fragility of CaP scaffolds (which are highly porous), pointing them out as not suitable for weight-bearing bone defects. Therefore, in order to improve CaP mechanical and structural properties, different combinations have been attempted by adding other components with viscoelastic properties (tolerating high levels of strain or deformation and able to fill irregular-shaped bone defects) such as collagen [27], alginate [28], chitosan [29,30], polylactic acid (PLA) [31], and polyglycolic acid [32], giving rise to injectable hydrogel systems. They are typically biocompatible due to their large water content, and less prone to provoke an immune response [33]. The hydrogel CaP scaffolds seem to be a suitable option for early tissue regeneration since they serve as a temporary matrix, providing mechanical stability and traction for migrating cells from adjacent tissues that gradually degrade the scaffold, replacing it with new bone. Attempts to develop ACP-based scaffolds have also been carried out, due to the fact that ACP particles are easily resorbed, releasing calcium and phosphate ions as they are required for new bone formation. However, since ACP is highly instable and tends to crystallize into brushite and HA minerals, the inhibition of this process has been addressed by generating an ACP hydrogel with PEG, plus the addition of both citrate and zinc, showing the latter the greatest stabilization [34]. This result paves the way for the future development of stable ACP scaffolds, which could be injected at the lesion site and function as a precursor material for new bone synthesis. 

Another noteworthy approach to improve scaffold biomechanical properties rely on the addition of metal traces such as strontium, which is naturally found in bone ECM [35,36] or non-naturals such us barium titanate [37,38]. Either one in combination with CaP composites seems to produce a good response regarding not only cellular adherence and proliferation, but promoting osteogenic differentiation. Barium titanate, similar to other solid materials (crystals, certain ceramics, or even bone itself), presents piezoelectric properties, meaning it accumulates electric charge in response to applied mechanical stress. Therefore, these types of materials can be deformed with physiological movements and consequently, provide an electrical stimulation to the tissue microenvironment, enhancing the tissue regeneration without any external source [39]. Several piezoelectric ceramics including potassium sodium niobate [40], lithium sodium potassium niobate [41], zinc oxide [42], or polymers such as polyvinylidene fluoride and PLA, are being studied to determine which material offers the best properties in terms of developing efficient electroactive prosthetic implants for bone repair [43,44].

Finally, the combination of CaP-based composites with different components of human bone tissue is also being explored. Over the last 20 years, autografts have been established as the gold standard in bone regeneration procedures, ensuring native structure and properties of bone ECM along with avoiding rejection from the immune system. However, the autologous bone supply is limited and the need to perform an additional surgery leads to the increased possibility of infections and donor site morbidity. The alternative focuses on using xenografts (usually from pigs or bovines [45,46]), or allografts from healthy donors [47,48,49]), which although solve the problem of availability, carry the risk of pathogen transmission and may induce the rejection by the recipient. Thus, a successful usage of allografts and xenografts in vivo requires a thorough removal of the component inducing the immune response such as elimination of the donor cells by decellularization [50,51] while maintaining the composition and functionality of ECM intact, vital for osteogenic induction [13]. Pulverized human bone and chitosan (a polysaccharide derived from chitin, a natural biopolymer) in combination with a β-TCP scaffold has been shown to promote cellular viability and osteogenic differentiation in vitro [52]. Even more, ALP activity was increased in the bone-containing sample compared to the control scaffold with only chitosan and CaP. Sargolzaei and coworkers assessed the effect of OCP granules and rat bone matrix gelatin (a polymer derived from the hydrolysis of collagen), alone or in combination, in critical-sized tibia defect in rats [53]. All three implants exhibited similar positive results, improving bone repair, and showing a good resorption of implanted materials in the early stages of bone formation. However, in the combinatorial scaffold, both type of particles, especially the bone matrix gelatin, were absorbed more rapidly compared to implants of each material alone, which could explain the lack of synergistic effect between OCP and bone matrix gelatin. The same study was performed in a rat mandibular defect model and the combination of OCP and bone matrix gelatin showed significantly better results than each material alone in terms of newly formed bone volume [54]. 

In addition to the composition of the material, the osteoinductive capacity of a scaffold designed for bone tissue engineering is highly dependent of the pore microarchitecture. Thus, high porosity and interconnectivity between the pores is essential not only for the correct transport of oxygen, nutrients, and essential factors, but to promote cellular infiltration and vascularization of the tissue. Scaffolds can have pores of different sizes ranging from macropores (>100 μm), which induce the cellular infiltration (such as macrophages to eliminate bacteria) and vascularization, to micropores (<50 µm). Osteoblasts, with an own size of 10–50 μm, prefer larger pores in the range 100–200 μm [55]. Even more, recent evidences have indicated that a bigger pore size (300–800 µm) leads to better osteoblast colonization, vascularization, and bone formation [56], accordingly with natural trabecular bone, which presents a pore size of up to 1 mm [57]. Besides, the morphology and porosity of the graft also influences the degradability and the mechanical properties of the implant. Therefore, when designing the pore size and distribution in a scaffold, it is also necessary to consider the degradability of the material, since high porosity and interconnectivity accelerates the degradation, compromising the mechanical and structural properties of the implant before it is completely substituted by new bone [57]. 

The simultaneous addition of micropores together with macropores in CaP-based scaffolds, improves bone growth in the macropores and provides them with better mechanical properties. New bone growth into the micropores improves the load transfer, decreases crack propagation and provides a toughening mechanism due to the chemical bond that forms between CaPs and bone [58]. The CaP-based materials enable a chemical bond between bone and scaffold through the formation of an apatite layer at the interface of both. Such a strong chemical bond in micropores, which are well-connected with macropores, provides a larger anchoring area that improves the stability and load transfer, resulting in better crack arrests. Definitely, both macro and micropores increase the total surface of the bone-scaffold interface leading to better mechanical integrity and osteointegration of the scaffold within the defect. Besides, micropores can induce capillary forces that enhance the cells to infiltrate and attach to the scaffold, promoting a homogeneous bone distribution [59]. The increased surface area can therefore offer more protein adsorption sites and accelerate the release of degradation products (calcium, strontium, or magnesium), which facilitate several cellular processes: attachment, proliferation, differentiation, biomineralization, etc. [60]. In agreement with this line, recently, it has been demonstrated that high microporosity (39%) indirectly enhances osteoconduction in wide-open porous CaP-based scaffolds [61]. The increased specific surface area facilitate bone ingrowth by increased Ca^2+^ ion release, which stimulate the cells for new bone synthesis.

In conclusion, the current trend in the field of tissue engineering focuses on the design of large-scale highly reproducible synthetic scaffolds, with CaP as a key component, which meets the properties that we have discussed, such as osteoconduction, osteoinduction, biocompatibility, and having a degradation rate equal to the new bone formation rate, so that it can be gradually replaced by host tissue. These composites can have different presentations, granules, scaffolds, or hydrogels, with different pore microarchitectures. Moreover, the combination of several materials and micropore sizes favors a synergy between the different components, enhancing the bone regenerative properties of the scaffolds, and compensating their possible weaknesses. Overall, these diverse materials can be further supplemented with active molecules to improve their osteoinductive capacity and promote faster bone healing, which will be discussed in the following section.

### 2.2. Supplemented Scaffolds 

During the healing process, bone ECM provides biophysical and biochemical support to the bone cells by dynamically interacting with osteoclasts and osteoblasts, regulating resorption and new bone formation. In that way, the composition and structure of inorganic and organic bone matrix may directly affect bone quality [13] and determine the fate of the progenitors of bone cells. Different strategies to closely mimic the bone microenvironment focus on adding bioactive factors to scaffolds [62,63]; as surface modification of scaffolds or via the addition of bioactive molecules and drugs that regulate bone tissue homeostasis. 

#### 2.2.1. Surface Modifications 

The attachment of a bioactive domain to the surface of the scaffold has been recently proposed as a strategy to improve cell adhesion, proliferation, and osteogenic differentiation of MSCs. We will now state several novel strategies such as silk fibroin (SF), hydrogels, and demineralized bone matrix (DBM), based on this approach. 

##### Silk Fibroin

SF, a fibrous protein produced by the domestic silk moth, *Bombyx mori,* is a promising natural organic material for use in biomedical applications, thanks to its biocompatibility and biodegradability properties. However, its weak gelation performance and the current lack of biochemical cues to trigger cell proliferation and differentiation, significantly limits its clinical application. To solve this problem, Yan Y. and collaborators developed novel hydrogels from SF containing abundant residues of RGD (arginine-glycine-aspartate tri-amino acid sequence; the most widely studied adhesive peptide in the biomaterials field [64]), which besides acting as cell adhesive peptides, are also responsible for signal transduction and osteogenic differentiation of MSCs [65,66]. Moreover, an improved version consisting of the addition of a small peptide gelator (NapFFRGD; Nap- phenylalanine- Phenylalanine-RGD) to the SF solution through cooperative molecular self-assembly resulted in a more stable SF hydrogel at a much lower gelation concentration plus much shorter gelation time [67,68]. 

Another novel strategy to improve the cell adhesion, proliferation and differentiation into SF scaffold is the adhesion of an elastin-like polypeptide (ELP, Val-Pro-Gly-Xaa-Gly) [69] via simple and green dehydrothermal (DHT) treatment, which represents an environment-friendly strategy and possesses high reproducibility [70,71]. Chen and coworkers demonstrated that bone marrow-derived MSCs (BM-MSCs) exhibited not only improved spreading and proliferation on the SF-ELP-DHT scaffolds, but also showed enhanced mature bone tissue formation compared to the naked SF scaffolds [72]. These results pointed out recombinant ELP modified silk scaffold as a promising candidate material for bone regeneration, given that it could mimic the required bone 3-dimensional (3D) microenvironment.

##### Hydrogel

Bioactive hydrogels have also been a focal point in the field of bone regeneration due to their ability to mimic the natural ECM microenvironment of the bone [73]. However, biopolymer-based hydrogels suffer from low mechanical properties, uncontrolled degradation, plus insufficient osteogenic activity, which limits their applications in bone regeneration. To overcome these drawbacks, hybrid gelatin/oxidized chondroitin sulfate (OCS) hydrogels have been developed as bioactive fillers [74]; while chondroitin sulfate is a glycosaminoglycan found in the bone ECM that increases the efficacy of arrangement of certain growth factors (GFs) involved in bone regeneration, gelatin, a water-soluble biocompatible biopolymer, facilitates cell adhesion and biomolecules deposition. Moreover, the incorporation of mesoporous (contains pores with diameters between 2 and 50 nm) bioactive glass nanoparticles (MBGNs) in the hydrogels significantly improve their mechanical properties, as has been demonstrated both in vitro and in vivo through the proliferation and osteogenic differentiation of rat BM-MSCs and rat cranial defect restoration, respectively. Therefore, the hybrid Gelatin-OCS/MBGN hydrogels is another interesting option to consider as injectable biomaterials or scaffolds for bone regeneration/repair applications. 

Other approaches that aim to recapitulate the complexity and signaling properties of bone ECM are focused on the development of microporous (pores smaller than 2 nm in diameter) and nanofibrous hydrogels exhibiting multiple bioactive epitopes [75]. The supramolecular environment is created by orthogonal enzymatic cross-linking that comprises hyaluronic acid modified with tyramine (derived from the amino acid tyrosine; HA-Tyr) and peptides amphiphiles (peptide-based molecules that comprises a hydrophilic peptide sequence attached to a lipid tail; PAs), designed to promote cell adhesion (RGDs-PA), osteogenesis (Osteo-PA), and angiogenesis (Angio-PA). Results confirmed the capacity of the HA-Tyr/RGDs-PA/Osteo-PA/Angio-PA hydrogel to promote cell adhesion as well as osteogenic and angiogenic differentiation. This strategy looks encouraging not only for bone tissue regeneration in vivo, but for lifelike bone tissue engineering in vitro. For instance, since the hydrogel recreates key structural and signaling elements of the native bone environment, in vitro drug screening could be a promising application. 

##### Demineralized Bone Matrix 

As mentioned before, DBM a polyporous bioscaffold commonly used for bone regeneration must be processed before being used for bone engineering purposes, losing its cell adhesion and osteoinductive abilities. Selective cell retention technology, based on the functionalization of DBM with molecules known to bind cells, has been used to improve the MSCs adhesion to the DBM and therefore the osteoinductive abilities of these scaffolds. Thus, DBM scaffolds with collagen-binding domains (CBD) have been recently designed, containing IKVAV (isoleucine-lysine-valine-alanine-valine) and RGD sequences, which are the core functional amino acid sequences of laminin and RGD-containing ECM proteins, respectively [76]. As expected, this DBM/CBD-IKVAV-cRGD composites increased the MSC adhesion capacity in vitro and osteogenesis in vivo. In this line, other scaffolds with the same approach have also shown promising results, such as a DBM scaffold with a CBD containing the core functional amino acid sequences of laminin α4 (CBD-LN peptide) [77]. In vivo, this DBM/CBD-LN scaffold promoted not only rapid bone formation but also angiogenesis, establishing its reputation as a new potential biomaterial in bone tissue engineering.

In addition to cellular adhesion and differentiation, the recruitment of a sufficient number of MSCs and ECs to the bone defect area is critical for bone repair. For instance, the regulation of protein tyrosine phosphatase 1B (PTP1B; a protein localized at the cytoplasmic face of the endoplasmic reticulum which is a negative regulator of the insulin signaling pathway) has been closely related to the stable residence of these MSCs and ECs in their niches. It has been suggested that the phosphorylation state of PTP1B tyrosine-152 (Y152) plays a central role in initiating the departure of MSCs and ECs from their niches and their subsequent recruitment to bone defects. In fact, the peptide 152RM (PTP1B Y152 region-mimicking peptide) loaded onto DMB scaffolds with mesoporous silica nanoparticles (MSNs) [78] significantly inhibited the phosphorylation of PTP1B Y152 [79], enhanced MSCs migration and osteogenic differentiation. Moreover, in vivo studies showed that this scaffold coupled the osteogenesis and angiogenesis processes, by inducing bone formation and the expansion of a certain type of blood vessels adjacent to the growth plate, closely related to the speed of bone healing [80]. 

#### 2.2.2. Addition of Bioactive Molecules 

As mentioned above, in addition to its structural role, ECM provides a complex network of biochemical and physiological signals that affect cellular proliferation and differentiation [81]. Although bone ECM is mainly composed by collagen type I, there have been identified more than 100 ECM proteins other than collagen type I [82]. For this reason, several approaches based on the addition of different bioactive molecules (such as hormones and GFs) to novel scaffolds have been carried out in order to promote osteogenic differentiation of MSCs and in consequence, bone formation [83]. 

MSCs are the common progenitors of osteoblasts and adipocytes; hence, it is not surprising that MSCs’ fate is delicately balanced and regulated by a number of signaling pathways involving different players. The identification of specific molecular switches that govern MSC lineage commitment has been crucial to promote osteogenic differentiation of MSCs. Tribbles homolog 3 (Trb3), a member of tribbles family pseudokinases, exhibits essential roles in cellular differentiation by regulating the activity of various transcription factors and GFs such as BMPs [84]. Since Trb3 stimulates osteoblastic differentiation in vitro and in vivo [85], Fan and coworkers designed a novel gelatin-conjugated caffeic acid-coated apatite/PLGA scaffold to induce its local delivery in vivo [86]. They demonstrated that Trb3 really acts as a key molecular switch determining MSC lineage fate, suggesting that it could be a treatment option to improve bone repair, by promoting osteoblastic commitment of MSCs at the expense of adipocyte differentiation. On the other hand, ECM remodeling has also been proposed as a novel strategy to control MSCs fate during self-healing, given that the regulation of the expression of matrix metalloproteinases (MMPs), metallopeptidases responsible for the cleavage of the protein components of ECM, may induce MSCs differentiation into osteogenic lineage. For instance, growth of MSCs on a remodeled Col I matrix by MMP13 stimulates osteogenic differentiation and self-healing of bone tissue [87]. 

Another compelling alternative focuses on bioactive materials containing hormones which regulate bone homeostasis. Parathyroid hormone (PTH) mediates calcium and phosphate homeostasis, thus regulating bone growth. In fact, the 1–34 amino acid fragment of PTH (PTH(1–34), also known as teriparatide), is the active sequence responsible for the bone remodeling function of PTH [88] and it has been approved for its use as an osteoanabolic drug in the clinical treatment of bone defects, such as osteoporosis [89]. PTH(1–34) along with nano-HA (nHA) and hydrogel combinations (to emulate the natural structures of bone) have been integrated to facilitate osteogenic differentiation of BM-MSCs [90]. The nanofibers and porous structure of the Gel-nHA-PTH scaffolds enhanced cell adhesion and showed good binding with bone tissue. Furthermore, with the PTH(1–34) addition, the scaffold nanofibers became finer, which increased its conducive to bone regeneration. Predictably, implantation of the hydrogel into a rat cranial defect model led to efficient bone regeneration, revealing the simultaneous therapeutic effect of nHA and PTH during the treatment process. 

At last, the combination of osteoinductive GFs with osteoconductive biomaterials remains a promising approach to promote bone regeneration [91]. GFs are the most influential bioactive molecules and mediators of the natural bone repair process. Although these soluble factors have approved applications in bone regeneration, they present several limitations that could restrict their clinical usage [92,93]. For instance, early GF delivery approaches [94] resulted in low availability of bioactive GFs due to their rapid degradation in vivo, short half-life in physiological conditions, and deactivation by enzymes [95]. In fact, the poor pharmacokinetics of these proteins has led to the delivery of high doses, with the consequent increase in the risk of serious side effects. To solve this problem, the development of novel vehicles able to control the release of GFs is the goal to be achieved [96]. 

##### BMP-2

Multiple GFs have been identified to be involved in bone regeneration, including platelet-derived growth factor, transforming growth factor-beta (TGFβ), fibroblast growth factors, insulin-like growth factors and BMPs. Among them, BMP signaling pathway, and in particular the signaling elicited by BMP-2, has been the most extensively studied due to its role in osteoblastic differentiation [97], angiogenesis [98], and cell signaling during fracture healing [99]. In fact, BMP-2 is considered the most remarkable bone-related GF due to its ability to increase the expression of osteogenic markers [100], such as ALP and osteocalcin [101], besides its role in the early stage of bone formation and repair [99].

However, these proteins are so potent that they can induce undesired bone formation in other tissues, and accordingly they require a vehicle to guide them to the damaged area [102]. For instance, products containing recombinant human BMP-2 (rhBMP2) [103] loaded in bovine absorbable collagen-type-I matrix scaffold have been used clinically to treat open tibia fracture [104], spine and craniofacial defects in the last decade [105]. These and other rhBMP2 based products, however, have shown controversial results in terms of efficacy and adverse effects [106]. Despite delivery of supraphysiological doses of BMP-2 being needed to induce bone formation, those doses seem to induce pathological events [107]. To cope with these limitations, intensive research studies are still ongoing in order to determine the best material carrier of BMP-2 [108], which can deliver the minimum required dose for improving bone repair and thus diminish side effects. To this aim, a large number of material carriers and delivery systems have been investigated for controlled, localized, and sustained release of BMP-2 [109,110]. 

Physiologically, BMP-2 bioavailability and signaling is regulated by either low or high binding affinity to ECM components [111]. In fact, some tissue-engineering strategies combine recombinant BMPs with naturally occurring ECM components (derived from MSCs [112]), in such a way that it modulates BMP-2 release and therefore enhances bone formation. For instance, Larochette and coworkers compared the efficacy of osteogenic mineralized MSC-derived ECM to the one obtained from ECM from undifferentiated hMSCs, using implanted polycaprolactone scaffolds [113]. The outcomes reflect that the osteoinductive potential of BMP-2 was greater when loaded within an osteogenic mineralized MSC-derived ECM, displaying a higher sequestration capacity of BMP-2 over time in vivo.

To improve the system, the encapsulation of BMPs into polymeric microspheres has emerged as one of the most promising methods to provide local and controlled delivery of BMP-2. However, fabrication of microspheres requires the use of toxic solvents which limits the bioactivity retention and their commercialization. To solve this problem, a method for solvent-free fabrication of porous microspheres from high internal phase emulsions using a controlled fluids setup (polyHIPE) has been developed [114]. In addition to the advantage of solvent-free fabrication, this method uniquely provides in-line loading of BMP-2 directly into the pores of the microspheres, with high loading efficiencies. Recently, key relationships between microsphere properties and the resulting BMP-2 release kinetics have been established [115]. First, bioactivity retention of encapsulated rhBMP2 was confirmed. Next, it was established that the BMP-2 release from microspheres induced osteogenic differentiation of hMSCs. Finally, the microsphere incorporation had minimal effect on the cure and compressive properties of an injectable polyHIPE bone graft. Overall, this work draws attention to the strong potential that these microsphere-polyHIPE composites present to enhance bone regeneration through controlled release of BMP-2 and other GFs. Moreover, the use of microspheres has demonstrated great advantages when compared with other BMP-2 delivery systems such as hydrogels and surface modified ceramics; typical mesh sizes of hydrogels result in a burst release that does not allow controlling kinetics, while surface-modified ceramics present reduced loading efficiencies during fabrication, which raises scale-up concerns.

Recently, spatiotemporal delivery of BMP-2, along with other factors that play an important role in bone formation, has been proposed to improve bone regeneration. While chemokines (such as Interleukin-(IL)-8) recruit circulating stem cells to the defect site [116], GFs such as BMP-2, induce the recruited cells to undergo chondrogenesis and osteogenesis to form bone [117]. That way, and according to the key steps of natural regenerative process, it is crucial to combine stem cell recruitment and bionic sequential delivery of chemokine and GFs to achieve effective bone regeneration. Therefore, the synergistic effect of BMP-2 and IL-8 on the key processes of bone regeneration was studied and then, a spatiotemporal delivery system for rapid in situ guided bone regeneration was designed [118]. Thus, macroporous (pores larger than 50 nm in diameter)/mesoporous bioactive glass scaffold has been used as matrix, to synergistically achieve a rapid release of IL-8 followed by a long-term sustained release of BMP-2. Outcomes demonstrated efficient stem cell recruitment and a “chondrogenic/osteogenic balance”, thanks to the spatiotemporal delivery of IL-8 and BMP-2. Ultimately the scaffold induced early extensive bone mineralization and an advanced regeneration throughout the repair of large bone defect. Overall, this new delivery system could provide insights toward designing bone-repairing biomaterials with higher regenerative efficiency.

Finally, multicell-mediated bone tissue regeneration has been studied by the use of rhBMP2-loaded trimodal macro/micro/nano-porous bioactive glass scaffold as a substrate model [119]. First, the combination of different porous structures regulates cellular function: while macropores activate migration of cells, micro/nano-scale pores increase the specific surface area generating expedited dissolution-deposition and rapid material biodegradation [120]. Then, the incorporation of BMPs lead to the stimulation of osteoclastogenesis as well as promoting osteogenesis, ensuing osteoclast-regulated material resorption [121,122]. That way, as results suggested, rhBMP2 facilitated osteoclastogenesis-mediated scaffold degradation and up-regulated osteogenesis. Synchronization of material resorption and new bone formation was vital to achieve harmonious bone regeneration in the treatment of large bone defects. 

#### 2.2.3. Addition of Drugs Relevant for Bone Tissue Homeostasis 

Some materials, in addition to enhancing the mechanical properties of natural polymers, overactivate osteoclasts and impair proliferation and osteogenic differentiation of MSCs; that is the case of the graphene oxide (GO)-related hydrogels [123,124]. To address this problem, administration of antiresorptive drugs such as bisphosphonates have been used to rebalance the general bone microenvironment and promote osteogenic differentiation. Hence, Alendronate (Aln), a first-line antiresorptive drug used in clinical treatment of osteoporosis, has been bound to GO-related type I collagen hydrogel, creating a Col-GO-Aln sponge [125] which exhibited active anti-osteoclastogenic and osteogenic ability in vitro and in rat preclinical models of osteoporosis. These results suggest the potential of GO related biomolecule loaded hydrogel in the treatment of osteoporotic bone defects.

Finally, the temporally controlled delivery of biochemical compounds has also been addressed with MSNs designing films that can guide MSCs differentiation towards the osteogenic lineage. These films have been loaded with dexamethasone, a glucocorticoid known to induce osteogenic differentiation of MSCs in vitro [126]. Temporally controlled dexamethasone delivery led to increased ALP levels and matrix mineralization compared to directly supplementing dexamethasone to the medium. Thus, MSN coatings mimic the sequential appearance of bioactive factors during tissue regeneration, which will ultimately lead to biomaterials with improved bioactivity.

**Figure 1:**
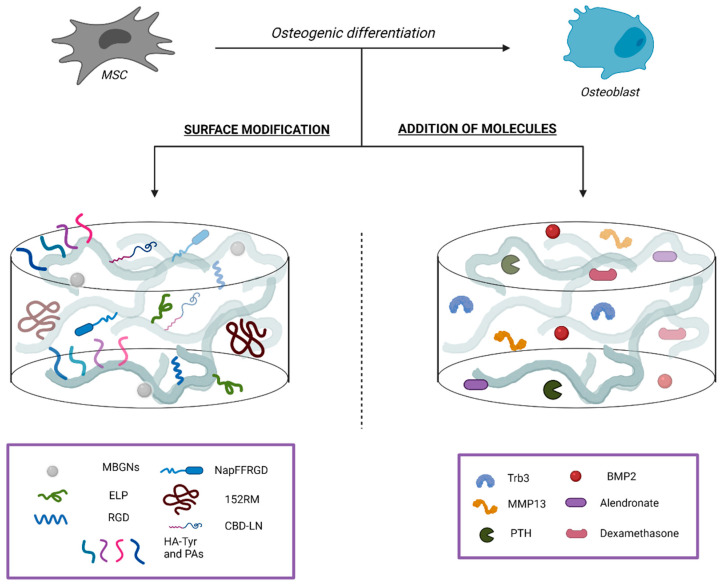
Regulation of cell fate and induction of osteogenic differentiation of MSCs by supplemented scaffolds. Surface modifications of the scaffolds by the attachment of a bioactive domain (**Left**), with the aim of improving adhesion, proliferation, and osteogenic differentiation of MSCs. Addition of bioactive molecules or drugs (**Right**) regulates bone homeostasis to emulate the complex network of biochemical and physiological signals that are representative in bone ECM.

The mentioned addressed approaches are summarized in Figure 1. 

### 2.3. Macrophages Polarization

In bone tissue engineering, osteointegration of the engineered graft is a key process occurring at the bone-implant interface, prompted by the response of the immune cells to the graft and the subsequent differentiation of osteoprogenitors. In fact, this immune reaction to the scaffolds is of great interest, since it is known to be a crucial factor influencing healing effectiveness. The first immune cell players interacting with bone implants are macrophages, orchestrating the host immune response to the grafted biomaterial. Bone repair can be divided into a first proinflammatory stage and a subsequent regenerative phase [127]. Immediately after a fracture has occurred, immune cells such as platelets, neutrophils, lymphocytes, and macrophages are recruited to the site of bone injury, playing a critical role in bone fracture repair by secreting inflammatory factors. Among them, macrophages and phagocytic cells differentiated from monocytes, take part in these two different stages of bone healing process, taking advantage of their functional plasticity, determined by the molecules they secrete. Thus, proinflammatory M1 macrophages are needed for the first stage of bone repair, facilitating the recruitment and osteogenic priming of MSCs to the injury site. Conversely, anti-inflammatory M2 macrophages, promote bone tissue healing [127]. This polarization of M1 macrophages to the M2 phenotype is a key step not only for bone healing but also for the osteointegration of bone tissue engineered grafts. In fact, chronic inflammatory conditions, such as diabetes, originate in an imbalanced host immune reaction to scaffold, in which the switch from M1 to M2 macrophages does not occur at the bone-implant interface, determining the failure of the tissue engineering graft [128]. Therefore, great efforts are currently being addressed to design immunomodulatory and, at the same time, pro-osteogenic scaffolds capable of regulating and boosting the switch of M1 macrophages to M2 phenotype. The use of pro-osteogenic scaffolds carrying immunomodulatory molecules such as ILs or micro ribonucleic acids (microRNAs) [129,130] or the modulation of surface topographical cues of the scaffolds [131] are among the strategies currently being used to improve the bone healing facilitated by endogenous macrophages.

#### 2.3.1. Interleukin-4

The combined use of a wide range of pro-osteogenic scaffolds such as decellularized bone matrix, bi-layer hydrogel-porous scaffolds, and calcium-enriched hydrogels [129,132,133] loaded with IL-4, a key anti-inflammatory cytokine secreted by M2 macrophages, is now being explored as a promising strategy for repair of bone defects [129,133]. Interestingly, calcium-enriched hydrogels loaded with IL-4 showed superior in vitro and in vivo abilities in inducing both M2 macrophages polarization and MSCs osteogenesis by enhancing TGF-β1/Smad pathway. The coordination of these two processes by the sustained release of IL-4 from scaffolds has been pointed out to be a key factor driving bone regeneration [129].

#### 2.3.2. MicroRNAs

MiRNAs, small non-coding ribonucleic acids (RNAs) involved in gene regulation at a post-transcriptional level, have been shown to be key players for the maintenance of bone tissue homeostasis by regulating both bone resorption and bone formation processes [134]. Indeed, a number of miRNAs with anti or pro-osteogenic capabilities have been identified, several of which are dysregulated in bone pathologies such as osteoporosis [135]. Due to the fact that miRNAs possess an intrinsic ability to target multiple genes and pathways, miRNA therapeutics (enhancement of the expression of miRNA with RNA mimics or miRNA expression inhibition by antagomiRs) is being considered as a coming realistic therapeutic strategy to elicit a more pronounced bone regeneration in bone-related pathologies. Since macrophages orchestrate a critical role in mediating host body reaction toward implanted biomaterial, the possibility of adding miRNAs therapeutics to pro-osteogenic scaffolds is being explored to induce M2 macrophage polarization [136].

In this way, the effectiveness of the inhibition of miR133a for bone repair has been recently tested in vivo by a bone tissue engineering approach with encouraging results [130]. Given the known role of miR133a as a negative regulator of osteogenesis in MSCs [137], Castaño and coworkers took advantage of collagen-nanoHA scaffolds loaded with antagomiR-133a, which was efficiently delivered to host cells. Moreover, a prominent bone repair in the antagomiR-treated group compared to the antagomiR-free scaffolds was confirmed by microstructure and histological analysis. Interestingly, an increase of M2 macrophages in the scaffolds loaded with antagomiR-133a was detected, suggesting a causative role of the increased presence of M2 macrophages in the scaffold interface with the accelerated bone healing observed in the antagomiR treatment group. Importantly, this study pointed to a new, understudied interplay between miRNA-mediated bone repair and M2 macrophage polarization which could be exploited in future scaffold-miRNA based strategies.

#### 2.3.3. Surface Topography Modulation

Modulating the surface topography of biomaterials to induce macrophage polarization has been a strategy widely studied over the last years [138]. Regarding bone-tissue engineering, the use of scaffolds with pore dimensions at the nanoscale level has been shown to be a pro-osteogenic strategy, by enhancing M2 polarization [139,140]. Recently, the underlying mechanism of how these nano-scale surface topographical cues modulate M2 polarization has been unraveled by transcriptomic approaches. By comparing honeycomb-like titanium dioxide (TiO_2_) structures with different pore sizes (ranging between 90 and 5000 nm), authors demonstrated the increased osteogenic potential of 90 nm pore scale scaffolds in vitro and in vivo, which enhanced MSCs osteogenic differentiation and M2 macrophage polarization [131]. Interestingly, the more pronounced confinement of macrophages in honeycomb-like TiO_2_ scaffolds with the smaller pore (90 nm) induced an activation of the RhoA/ROCK signaling pathway linked to an increased formation of filopodia, a mechanism pointed to be the driving cue shifting macrophages toward M2 polarization. 

## 3. Strategies Promoting Bone Healing through Exogenous Response 

### 3.1. Cellular Therapies

Cell therapies based on MSCs have been investigated to treat a number of diseases with the aim of restoring the homeostasis of target tissues. In the context of bone regeneration and taking into account that MSCs are the natural progenitors of osteoblasts, the beneficial effects of MSCs administration have been attributed to different, not mutually exclusive mechanisms: multipotency, immunomodulatory potential, and trophic effects [141]. MSCs from several sources have been utilized in the field of bone regeneration including bone marrow (BM), adipose tissue, umbilical cord, and recently, dental-related tissues that are normally discarded [142]. In order to benefit from the properties of the MSCs, it is necessary to achieve a high number of them, so the isolation and expansion of these cells are crucial factors. Considering that the MSC doses for bone disease range from 1 up to 350 million cells per dose [143], and that the patient may need multiple infusions, the amount of MSCs needed is quite significant. 

As well as having the required number of cells, it is crucial to ensure that they maintain their stemness. During ex-vivo expansion, cells are subjected to deleterious aging effects, which could compromise their ability to differentiate into different cellular types and limit their clinical application. In this context, several encouraging attempts are underway such as the use of melatonin to preserve the stemness of BM-MSCs during long-term passaging [144] and promoting MSCs-driven local bone regeneration in both ectopic sites and critical-sized calvarial bone defects. Melatonin exerts its protective effect by its great antioxidant capacity [145,146], acting as an intracellular signaling regulator [147], delaying senescence [148], and promoting ossification [149,150]. 

Other strategies to improve MSCs-based therapies performance have focused on the influence of the recipient microenvironment. Increasing evidence strongly suggests that cells respond to the needs of the microenvironment in which they are found. For instance, a recent study claimed that MSCs secretome seemed to address the primary need of the cell environment, presenting an immunomodulatory or anabolic MSC-derived secretome protein composition depending on the environmental pathological state [151]. Accordingly, it is not surprising that pre-conditioning of MSCs has shown to be effective in resisting recipient pathogenic microenvironmental impacts and improving regenerative potential [152,153,154,155]. 

Taken together, it is of great significance to explore safe and effective reagents that could help to improve the current efficiency of cellular therapies, enhancing MSCs proliferation and differentiation, along with cells survival after transplantation.

Despite MSCs having many properties that makes them promising candidates for bone repair therapies, clinical translation has been slower and more challenging than desired. A great effort is being carried out in this field, evidenced by the large number of clinical trials that are being conducted (clinicaltrials.gov, accessed on 20 July 2021), to better understand the mechanisms that are decisive for the success of the treatment, making it possible to better define the optimal parameters in each case and to standardize the entire process for better reproducibility. Cellular heterogeneity of MSCs seems to be one of the weaknesses resulting in often variable outcomes [156]. In addition, there are many points that should be taken into account throughout the entire process, from the collection of the cells to their administration, which can be the reason for this observed high variability. For example, the origin of the tissue from which the MSCs are obtained, the methods and route of administration (local or systemic), the amount of MSCs that are inoculated in each dose, the number of doses per patient and the need or not to pretreat the cells to improve their viability. Despite all these handicaps, we cannot overlook the promising results from recent clinical trials addressing bone pathologies treatments based on MSCs therapy [157,158,159,160]. This is the case of TERCELOI, a phase I clinical trial (code NCT02172885), which demonstrated the safety and feasibility of repetitive MSCs infusions in two pediatric patients affected by Osteogenesis Imperfecta, a rare, genetic disease characterized by extremely low bone mass and increased fracture risk. During the clinical trial, both patients showed a reduction in bone fractures, as well as improvements in bone-related parameters and quality of life. Moreover, the study of the mechanism of action indicated a pro-osteogenic paracrine response in patients’ serum as a consequence of cell infusions, thus, reinforcing the hypothesis that MSCs elicit their beneficial effects, at least in part, through paracrine mechanisms [161]. 

MSCs release extracellular vesicles (EVs), including exosomes (derived from early endosomes, 50–200 µm) and microvesicles (originated from plasma membrane, >200 µm). These EVs mediate cell-to-cell communication, thus altering cell or tissue homeostasis at short or long distances in the body. They contain a plethora of bioactive molecules and remarkably, these EVs have shown results very similar to MSC transplantation in many cases (the so-called “cell-free” therapy) [162]. Therefore, currently there is a growing trend towards the use of MSCs as factories for EVs [163]. Recently, the systemic injection of EVs obtained from human urine-derived stem cells (USCs) has shown to effectively alleviate bone loss and maintain bone strength in osteoporotic mice by enhancing osteoblastic bone formation and suppressing osteoclastic bone resorption [164]. The molecules responsible for this improvement were collagen triple-helix repeat containing 1 and osteoprotegerin proteins, which were found to be enriched in USC-derived EVs.

In this line, exosomes from MSCs in late stages of differentiation presented a set of miRNAs related to osteogenic pathways that were able to induce osteogenic differentiation and mineralization of MSCs in vitro and in vivo [165]. In fact, MCSs treated with exosomes derived from BM-MSCs in vitro, have showed significantly upregulated osteogenic genes (collagen 1 (*COL I*), *ALP*, osteocalcin (*OCN*), and osteopontin *(OPN)*) together with angiogenic genes (vascular endothelial growth factor (*VEGF*), angiopoietin 1 (*ANG1*), and angiopoietin 2 (*ANG2*)). Considering that vascularization is a key step in bone repair, the promotion of angiogenesis by exosomes enhances the process of bone regeneration. Other tissues could also benefit from the use of exosomes as a cell-free therapy. 

### 3.2. Combinatorial Therapies of MSCs with Composites

Bone injuries involving a significant lack of bone, such as critical-size or nonunion injuries, need physical support that can serve as an anchor, able to guide the regeneration of the new bone. Therefore, in these cases, scaffolds providing support for the correct repair of the bone lesion endorse cellular administrations. Jungbluth and colleagues [166] have evaluated the efficacy of the combination of CaP scaffolds with induced pluripotent stem cell-derived MSCs (iMSCs) and autologous BM concentrate in a mini pig tibial defect model. Both, iMSCs and BM concentrates, in combination with CaP, showed an improvement in bone volume recovery of around 50% compared to the empty scaffold. Still, neither treatment matched the performance of autograft bone, being the most effective treatment with a volume of new bone formation of almost 80% [166]. 

Regarding hydrogels, most of the research on stem cell delivery has focused on methods involving cell encapsulation within a nanoporous mesh. However, recent researches suggest that the restrictive nature of this microenvironment significantly alters cellular behavior [167]. To solve this problem, microporous annealed particle (MAP) hydrogels have been proposed. Thus, cells can be incorporated during microgel annealing into MAP hydrogels, rather than being embedded in a nanoporous polymer mesh as occurs in conventional hydrogels. Cells in MAP hydrogels interact with the microgel surfaces but they are not encapsulated, claiming several studies the superior cell spreading in microgel-based scaffolds [168]. 

Spreading and mechanosensing activation of hMSCs incorporated in PEG-based MAP hydrogels can be modulated by tuning the modulus of the microgel particle building blocks [169]. Moreover, the effects of degradability and functionalization with different integrin-binding peptides on cellular responses has been explored [170]. The effects of a cyclized RRETAWA peptide (henceforth referred to as c(RRETAWA)), which targets α5β1 integrins and induces hMSC osteogenic differentiation [171], have been contrasted to the widely used RGD motif that binds to many different integrins. In brief, the outcomes have demonstrated that c(RRETAWA) functionalization increases osteogenic protein expression by hMSCs compared to RGDs-functionalized MAP hydrogels.

On the other hand, different metabolic pathways are becoming better understood, as well as the genes that govern the entire process of bone regeneration. Knowledge is taken into account by the new approaches to facilitate faster and more efficient healing. Thus, in addition to using scaffolds and MSCs, it is intended to add other factors that generate a favorable microenvironment for prompting fracture healing, as is the case of BMP-2. However, limitations on the doses needed and their stability is another difficulty to overcome by these attempts, as was disclosed earlier (BMP-2). Several interesting attempts have been made in recent years to develop approaches that allow a sustained and more physiological BMP-2 release. Kong and collaborators designed a system for delivering encapsulated allogenic BM-MSCs and BMP-2 into the fracture site [172]. BMP-2 was loaded into PLA microspheres, while BM-MSCs were encapsulated, together with BMP-2 containing microspheres, into sodium alginate microcapsules. The microencapsulation technology enables to enclose the cells within polymeric materials that function as a semi-permeable barrier, allowing the diffusion of nutrients, oxygen, and GFs, but preventing the host´s immune cells and antibodies to enter. The encapsulated allogenic BM-MSCs retained high viability and differentiation capacity, whereas encapsulated BMP-2 displayed a sustained release profile and maintained its bioactivity after microcapsule incorporation. Indeed, combined delivery of encapsulated BMP-2 and BM-MSCs greatly enhanced osteogenesis in a rat calvarial defect model. Considering that sources of autologous BM-MSCs are limited, this system seems to be promising since it allows the use of allogenic MSCs with very low immunogenic effect in the host.

Another approach that enables BMP-2 production over a period of several weeks is gene therapy by delivering nucleic acids encoding BMP-2 (either deoxyribonucleic acid (DNA) or RNA) to obtain controlled and sustained protein expression at the fracture site [173,174]. These strategies consist of both viral and non-viral methods, the latter being the preferred one for bone regeneration as it is considered safer (avoids immunogenicity and integration of the host genome). Non-viral gene delivery is often performed using plasmid DNA (pDNA); these circular, small, double-stranded DNA structures are stable, can be readily produced in bacteria and customized with a variety of different promoters [175]. However, the drawback focuses on the lower efficiency that this technique shows compared to viral methods. The resulting low amounts of transgene expression in the target area have made clinical translation quite challenging. Loozen and coworkers explored the conditions for optimal non-viral BMP-2 transgene expression, demonstrating the convenience of MSCs co-seeding in this procedure. The construct consisted of an alginate hydrogel containing cells and porous biphasic CaP granules loaded with pDNA of BMP-2 gene. The alginate-based scaffolds are quite popular for gene delivery therapies as alginate, a natural polysaccharide extracted from brown seaweeds, presents a porous structure and high water-absorption capacity, making it ideal matrix for cells encapsulation. In addition, it functions as a delivery vehicle for genetic material to cells. Alginate forms condensed complexes with DNA, protecting it from nucleases and facilitates DNA release in a timely manner, which results in a continuous controlled presence of it. Concerning DNA delivery, the construct performed satisfactorily; up to 50% of the initially loaded pDNA was released in the first three days, which increased to more than 60% after two weeks in vitro [176]. Despite these encouraging preliminary results, no bone formation was observed in the implanted areas, indicating that BMP-2 expression was still insufficient to promote osteogenic metabolism. 

In an endeavor to develop new strategies overcoming these poor outcomes, García-García, and collaborators [177] have proposed post-transcriptional gene silencing in MSCs via locked nucleic acid antisense oligonucleotides. The authors proposed the silencing of *Smurf1*, an inhibitor of BMP signaling pathway, to enhance MSCs differentiation and therefore, bone formation. To avoid using high levels of antisense oligonucleotides, they used a nontoxic lipid-based delivery system that promotes the intake by the cells through endocytosis. *Smurf1* silenced MSCs were encapsulated in alginate hydrogel scaffold along with BMP-2 microspheres. The in vivo implantation of this scaffold in an osteoporotic rat calvaria defect model showed a bone repair rate significantly higher compared to scaffolds containing BMP-2 or unsilenced MSCs, separately or in combination. In addition, the subcutaneous ectopic implantation in mice of the scaffolds containing MSCs were able to form bone tissue when seeded with a certain amount (6 μg) of BMP-2. Interestingly, scaffolds with half the dose of BMP-2 (3 μg) were able to elicit the formation of a mature and mineralized bone matrix only when they were seeded with MSCs in which *Smurf1* expression had been silenced. Therefore, *Smurf1* silencing increases the susceptibility of MSCs to BMP-2, allowing a significant reduction of the dose needed to achieve a therapeutic effect [177]. 

Another recent study tried a similar approach by using genetically modified MSCs (that constitutively overexpress BMP-2) incorporated into a chondroitin sulfate glycosaminoglycan injectable hydrogel scaffold to heal a critical size femur defect in rats [178]. Histological characterization revealed a lamellar-like structure indicative of mature bone in 12 weeks. Surprisingly, the bone maturity outcomes did not translate into functional differences in terms of mechanical strength and stiffness of the regenerated femurs. 

Bone fracture repair is an intricate process that implies a complex net of factors in a time order, tightly orchestrated. Understanding all these mechanisms will lead us to point out key regulators that could be targeted for a desirable response. Genetic engineering strategies combined with materials that support bone growth and promote MSC differentiation are presented as a powerful tool to promote bone healing; yet, a deeper knowledge regarding their safety and efficacy are required prior its clinical application.

### 3.3. Perfusion Bioreactors 

As mentioned above, bone surrogates based on MSCs, the progenitors of osteoblasts in bone, cultured in scaffolds have emerged as an exciting approach to directly repair bone defects or engineer bone tissue for transplantation. The latest research in this field points to dynamic 3D MSCs cultures by the use of bioreactors, which control physical parameters (pH, temperature, shear stress, oxygen and nutrient supply, waste removal) to achieve the maximum osteogenic potential of the engineered bone tissue. Thus, bioreactors of different types; spinner flasks, rotating bioreactors, and perfusion-based systems, have been widely used in in vitro and in preclinical studies to increase the osteogenic potential of MSCs cultured in a wide range of scaffolds [179]. These devices enable the 3D seeding and culture of MSCs under dynamic experimental conditions that mimic more efficiently the microenvironment of bone tissue than standard 2D cultures do. Perfusion bioreactors have been shown to be the most suitable for bone tissue engineering purposes; they provide a homogeneous distribution of cells and nutrients throughout the entire volume of the scaffold besides simulating bone interstitial fluid flow (the movement of fluids through the porous, mineralized ECM of bone). Thus, perfusing medium at high flow velocities has been shown to be essential for mimicking the bone interstitial fluid flow, which physiologically is mainly induced by the mechanical loading supported by the skeleton [180,181,182]. 

Currently, the most innovative strategies used in bone tissue engineering are aimed at the in vitro development of analogs of bone tissue, faithfully resembling the compositional and structural features of bone tissue, and therefore achieving more effective functional capabilities. In this line, the use of perfusion bioreactors in combination with two innovative cell culture approaches; culture of niche-relevant cells and scaffold functionalization, have achieved promising bone tissue engineered constructs. 

#### 3.3.1. Culture of Different, Niche-relevant Cell Types

The generation of functionally relevant bone tissue analogs by using perfusion bioreactors, which reproduce different cell compartmentalizations, is at the forefront of bone tissue engineering. In this complex process, a key step is the culture of different tissue-relevant cell types, which can be added sequentially or simultaneously into a 3D scaffold, to resemble the cell microenvironment of the specific target tissue to be studied and/or treated [183,184]. Thus, sequential cell seeding has been recently addressed to generate in vitro an analog of BM, a tissue mainly composed of cells with hematopoietic and osteoblastic lineages [184]. Taking into account these two cellular components of BM, the first MSCs were perfused and cultured in a HA scaffold under osteogenic conditions, and hence obtained osteoblasts plus the ECM secreted in the process of osteogenic differentiation. Once having generated the stromal component of BM, the hematopoietic component was introduced into the scaffold by perfusing hematopoietic progenitors isolated from human umbilical cord blood. The achieved in vitro BM tissue recapitulated the composition and structure of native human BM, demonstrating that this cell culture system enables the design of functional advanced tissue engineered construct, reflecting more closely the microenvironments of native tissues. In this line, a cartilage-bone engineered tissue for temporomandibular joint regeneration has been recently developed, in this case performing a co-culture of chondrogenic and osteogenic cells taking advantage of an ingenious dual perfusion system [183]. Thus, porcine chondrogenic and osteogenic progenitors were simultaneously seeded into independent compartments from a decellularized trabecular bovine bone scaffold. Each cell type was cultured under its specific requirements, such as differentiation medium (cartilage or osteogenic medium) and shear stress conditions (low shear to promote cartilaginous tissue and high shear to enhance osteogenesis). When tested in vivo, the resulting 3D cartilage-bone engineered tissue, which as expected resembled more reliably the physiological structure and composition of joint, demonstrated a greater capacity to restore the functionality of the jaw in minipigs, compared to bone only-engineered grafts generated under the same culture conditions [183]. 

#### 3.3.2. Functionalization of Scaffolds with ECM Proteins

The presence of ECM proteins in the scaffolds points to be a key element to enhance the regenerative potential of engineered bone tissues in perfusion systems [185]. Interestingly, two approaches, endogenous ECM generated by seeded cells or exogenously added ECM to scaffolds at the time of perfusing the cells, have been shown to enhance the osteogenic potential of engineered scaffolds [184,186,187]. Thus, by using perfusion bioreactors, the osteogenic preconditioning of MSCs in the scaffolds achieved a robust osteogenic response, both in vitro and in vivo, due to the presence of osteogenic ECM proteins secreted by seeded cells [184,187]. In fact, the simultaneous addition of cell-secreted ECM and BM aspirates (BMAs) into perfused scaffolds significantly increased the cell seeding efficiency when compared to naive scaffolds [186]. Interestingly, ECM-coated scaffolds also have been shown to promote greater vascular infiltration in vivo [186]. Osteogenic preconditioning of ECM-coated grafts perfused with BMAs increased blood vessels development correlating with a higher osteoanabolic capacity of these tissue engineered constructs, as expected [187]. Since poor vascularization of bone tissue engineered grafts after implantation has been traditionally a major drawback [188], the strategy of functionalizing scaffolds with ECM proteins to promote blood vessels infiltration into the scaffolds emerges as a real option that deserves further investigations. Moreover, the different cell-type seeding strategies aforementioned enables the possibility of seeding ECs precursors along with osteoprogenitors in perfusion bioreactors, opening the possibility of introducing vascular networks within the bone engineered scaffolds. 

## 4. Conclusions

**Figure 2:**
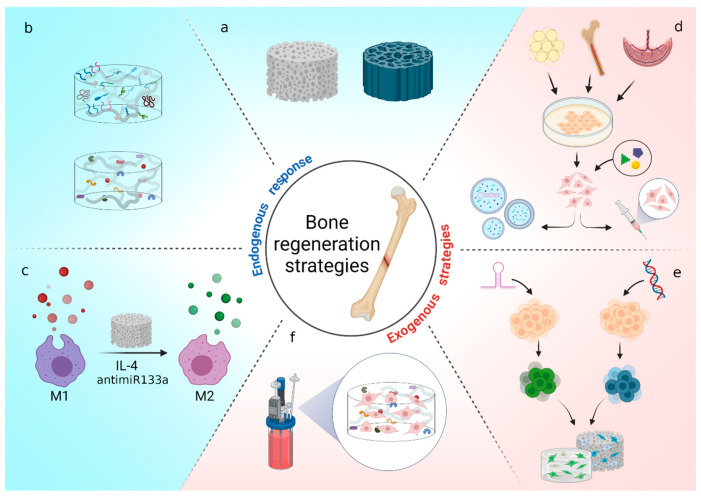
Schematic representation of different strategies for bone regeneration described in this review. The strategies are divided into two main categories; the ones promoting the response of endogenous cells to form new bone (**a**–**c**); and the ones that rely on the addition of MSCs or their derivatives (extracellular vesicles) to induce the bone healing (**d**–**f**). (**a**) Scaffolds can be obtained from bone grafts (autographs, allografts, or xenografts) or made of different natural and synthetic materials that promote bone regeneration. The properties of these scaffolds can be enhanced by the addition of surface modifications or soluble molecules such as proteins or GFs that improve their osteogenic effect (**b**). Macrophages polarization of pro-inflammatory M1 into anti-inflammatory M2 is a key step not only for bone healing but also for the osteointegration of bone tissue engineered grafts (**c**). Regarding strategies that depend on exogenous cells, MSCs from many origins (adipose tissue, BM, umbilical cord and placenta), must be harvested and expanded in order to obtain the needed amount of cells. Cells pretreatment prior its implantation in the patient could enhance their effectiveness. Likewise, the secretome of MSCs can also be used in the so-called “cell free” therapy (**d**). Other strategies that combine MSCs and composites try to promote a more efficient response by introducing genetic modifications into the cells by gene delivery systems (**e**). Lastly, bioreactors provide a better control of physical parameters to achieve the maximum osteogenic potential (**f**).

Despite the intrinsic capacity of bone for self-repair, the regeneration of large bone defects and especially under certain conditions such as aging and/or disease is a clinical challenge demanding novel osteoanabolic solutions. In consequence, different and very promising approaches, some of them still in an early development stage, are under extensive research, such as the boosting of endogenous bone resident cells and the exogenous addition of cells (usually MSCs) alone or in combination with a wide range of tuned scaffolds and/or pro-osteogenic molecules to drive bone regeneration (Figure 2). Strikingly, the recent development of functional bone tissue analogs, recreating the bone niche composition and structure by tissue engineering techniques entails a step closer to bone regeneration goal. Undoubtedly, in the near future, the development of next generation bone surrogates will be decisive in the success of bone healing. 

## Data Availability

Not applicable.

## Abbreviations

**Abbreviation**	**Definition**
3D	3-dimensional
ACP	Amorphous calcium phosphate
Aln	Alendronate
ALP	Alkaline phosphatase
BM	Bone marrow
BMA	Bone marrow aspirates
BM-MSCs	Bone marrow-derived MSCs
BMP-2	Bone morphogenetic protein 2
CaP	Calcium phosphate
CBD	Collagen-binding domain
DBM	Demineralized bone matrix
DHT	Dehydrothermal
DNA	Deoxyribonucleic acid
ECM	Extracellular matrix
ECs	Endothelial cells
EVs	Extracellular vesicles
GFs	Growth factors
GO	Graphene oxide
HA	Hydroxyapatite
HA-Tyr	Hyaluronic acid modified with tyramine
hMSCs	Human MSCs
IL	Interleukin
iMSCs	Induced pluripotent stem cell-derived MSCs
MAP	Microporous annealed particle
MBGNs	Mesoporous bioactive glass nanoparticles
miRNAs	MicroRNAs
MMPs	Matrix metalloproteinases
MSCs	Mesenchymal stem cells
MSNs	Mesoporous silica nanoparticles
nHA	Nano-hydroxiapatite
OCP	Octacalcium phosphate
OCS	Oxidized chondroitin sulfate
PAs	Peptides amphiphiles
PC-1	Plasma cell glycoprotein 1
pDNA	Plasmid DNA
PEG	Polyethylene glycol
PLA	Polylactic acid
PPi	Pyrophosphate
PTH	Parathyroid hormone
PTP1B	Protein tyrosine phosphatase 1B
rhBMP2	Recombinant BMP-2
RNA	Ribonucleic acid
SF	Silk fibroin
TCP	Tricalcium phosphate
TGFβ	Transforming growth factor-β
Trb3	Tribbles homolog 3
USCs	Urine-derived stem cells
